# Engineering Ultra-Low Thermal Conductivity in (Pb_0.8_Ge_0.2_Te)_0.95-*x*_(PbSe)_0.05_(PbS)*_x_* Quaternary Lead Chalcogenides Through PbS-Induced Phase Segregation

**DOI:** 10.3390/ma18143232

**Published:** 2025-07-09

**Authors:** Dianta Ginting, Hadi Pronoto, Nurato, Kontan Tarigan, Sagir Alva, Muhamad Fitri, Dwi Nanto, Ai Nurlaela, Mashadi, Yunasfi, Toto Sudiro, Jumril Yunas, Jong-Soo Rhyee

**Affiliations:** 1 Master of Mechanical Engineering Program, Faculty of Engineering, Universitas Mercubuana, West Jakarta 11650, Indonesia; hadi.pranoto@mercubuana.ac.id (H.P.); nurato@mercubuana.ac.id (N.); kontan.tarigan@mercubuana.ac.id (K.T.); sagir.alva@mercubuana.ac.id (S.A.); muhamad.fitri@mercubuana.ac.id (M.F.); 2 Physics Education Department, Syarif Hidayatullah State Islamic University Jakarta, Jl. Ir. H. Juanda No. 95, Cempaka Putih, Jakarta 15412, Indonesia; dwi.nanto@uinjkt.ac.id (D.N.); ai.nurlaela@uinjkt.ac.id (A.N.); 3 Research Center for Advanced Material, National Research and Innovation Agency, South Tangerang 15310, Indonesia; mashadi@brin.go.id (M.); yunasfi@brin.go.id (Y.); toto009@brin.go.id (T.S.); 4 Institute of Microengineering and Nanoelectronics (IMEN), University Kebangsaan Malaysia, Bangi 46300, Malaysia; jumrilyunas@ukm.edu.my; 5 Department of Applied Physics, Institute of Natural Sciences, Kyung Hee University, Yongin 17104, Republic of Korea

**Keywords:** thermoelectric materials, PbTe-based alloys, phase segregation, thermal conductivity, bipolar conduction

## Abstract

The shortage of tellurium and toxicity of lead are major obstacles to scaling mid-temperature thermoelectric generators. We engineer quaternary lead chalcogenides with composition (Pb_0.8_Ge_0.2_Te)_0.95-_*_x_*(PbSe)_0.05_(PbS)*_x_* (0 ≤ *x* ≤ 0.25), where Pb is lead, Ge is germanium, Te is tellurium, Se is selenium, S is sulfur, and *x* denotes the molar fraction of lead sulfide (PbS). The primary novelty lies in achieving ultra-low thermal conductivity through controlled phase segregation induced by systematic PbS incorporation. X-ray diffraction analysis reveals single-phase solid solutions up to *x* ≈ 0.10, with secondary PbS precipitates forming beyond this threshold. These PbS-rich phases create hierarchical microstructures that scatter phonons across multiple length scales, suppressing total thermal conductivity to 0.6 Wm^−1^K^−1^ at *x* = 0.15—approximately 84% lower than pristine lead telluride (PbTe) and approaching glass-like thermal conductivity values. Electrical transport measurements demonstrate sulfur’s role as an electron donor, enabling carrier-type control from p-type to n-type conduction. Despite moderate electrical power factors, the optimized composition (*x* = 0.20) achieves a peak dimensionless figure of merit *ZT* ≈ 0.34 at 650 K. This work demonstrates an effective strategy for tellurium-lean, lead-reduced thermoelectric materials through sulfur-induced phase segregation, providing practical design guidelines for sustainable waste heat recovery applications.

## 1. Introduction

The global energy crisis, characterized by increasing demand and diminishing conventional resources, has intensified the search for sustainable energy solutions. In this context, thermoelectric materials have emerged as promising candidates for direct waste heat recovery, offering a pathway to enhance energy efficiency across various sectors. Notably, approximately 60–70% of the energy produced worldwide is lost as waste heat during industrial processes, power generation, and transportation, presenting a significant untapped resource for sustainable energy harvesting [1,2]. Thermoelectric devices, which directly convert temperature gradients into electrical energy through the Seebeck effect, represent a solid-state solution with unique advantages including reliability, scalability, and zero emissions [3,4].

The conversion efficiency of thermoelectric materials is quantified by the dimensionless figure of merit (*ZT*), which can be calculated by [5,6]

(1) $$ZT=\frac{S^{2}\sigma T}{\kappa_{T}}$$

where *S* is the Seebeck coefficient (thermopower), *σ* is the electrical conductivity, *T* is the absolute temperature, and *κ_T_* is the thermal conductivity comprising both electronic (*κ_e_*) and lattice (*κ_L_*) contributions [5,6]. The Seebeck coefficient represents the magnitude of voltage generated per unit temperature difference across a material, fundamentally describing a material’s ability to convert thermal energy into electrical energy. A larger absolute value of the Seebeck coefficient indicates a more efficient conversion of temperature gradients into electrical voltage, making it a critical parameter for thermoelectric performance optimization [5,6]. Engineering high-performance thermoelectric materials necessitates the challenging task of simultaneously optimizing these interdependent transport parameters. Among various strategies, reducing thermal conductivity has proven particularly effective, as it directly enhances *ZT* without necessarily compromising electrical properties [7,8].

Understanding the temperature dependence of thermoelectric transport parameters is crucial for optimizing material performance across operational temperature ranges. In degenerate semiconductors, the Seebeck coefficient typically exhibits characteristic temperature-dependent behavior that reflects the underlying electronic structure and carrier scattering mechanisms. For p-type degenerate semiconductors, the Seebeck coefficient generally decreases with increasing temperature due to thermal broadening of the carrier distribution in the valence band. It then leads to a reduced energy dependence of scattering processes and consequently decreases the overall thermopower. This fundamental behavior must be distinguished from other temperature-dependent phenomena such as bipolar conduction. Bipolar conduction, where minority carrier activation at elevated temperatures can cause dramatic changes in transport properties [5,6,7,8], occurs when thermal energy becomes sufficient to generate electron–hole pairs across the band gap, creating intrinsic carriers that coexist with the dominant extrinsic carriers. This dual-carrier transport can lead to dramatic sign changes in the Seebeck coefficient from positive (p-type) to negative (n-type) or vice versa, as the minority carrier contribution becomes increasingly substantial due to differences in carrier mobility or density of states. At elevated temperatures, while one carrier type may dominate at lower temperatures, the contribution of the opposite carrier type becomes significant. It is eventually potentially surpassing the original majority carriers and leading to a net reversal in the sign of the Seebeck coefficient. Understanding and controlling bipolar conduction is essential for optimizing thermoelectric performance across wide temperature ranges. This phenomenon typically reduces the Seebeck coefficient magnitude and increases thermal conductivity through additional bipolar thermal transport contributions [5,6,7,8].

Lead chalcogenide (PbTe)-based compounds have demonstrated exceptional potential as mid-temperature (400–800 K) thermoelectric materials. It happens due to their favorable band structure, intrinsically low thermal conductivity, and high carrier mobility [9,10]. However, the widespread application of these materials faces significant challenges related to material sustainability and environmental concerns. Tellurium (Te), a critical component in PbTe systems, is an extremely rare metalloid—approximately eight times rarer than gold. This scarcity poses substantial supply risks for large-scale thermoelectric applications, with tellurium showing the highest market risk score (60 out of 100) among critical raw materials [11]. Furthermore, lead toxicity presents serious environmental and health concerns, causing neurological, developmental, and reproductive harm in humans, along with the contamination of soil and water sources [11].

To address these sustainability challenges, selenium (Se) and sulfur (S) have emerged as promising alternatives to tellurium due to their greater abundance in the Earth’s crust and significantly lower costs [12]. Recent research has demonstrated that quaternary PbTe-PbSe-PbS systems can achieve thermoelectric performance superior to binary and ternary ones. The thermoelectric performance leads chalcogenides while reducing the dependence on expensive tellurium [12]. The partial replacement of Te with Se and S creates high-contrast atomic mass fluctuations that effectively scatter phonons across multiple length scales. It then results in dramatically reduced lattice thermal conductivity [12,13,14,15].

The integration of germanium (Ge) into the PbTe matrix has drawn significant attention due to its ability to induce beneficial band structure modifications through band convergence effects [8]. Jiang et al. demonstrated that entropy engineering in chalcogenide systems can promote exceptional thermoelectric performance through both electronic structure optimization and thermal transport modulation [16]. Additionally, Zhu et al. showed that multiple valence band convergences combined with strong phonon scattering mechanisms leads to a remarkable enhancement in thermoelectric performance in p-type PbSe [17].

The development of quaternary lead telluride–lead selenide–lead sulfide (PbTe-PbSe-PbS) systems represents a strategic approach to address tellurium scarcity and reduce lead content while maintaining high thermoelectric performance. Introducing selenium and sulfur into the PbTe lattice creates a complex solid solution that facilitates multiscale phonon scattering through mass fluctuation, strain field effects, and dislocations [18,19]. Shih et al. (2025) recently demonstrated how interface engineering with two-dimensional materials can further enhance phonon scattering and electron transmission in chalcogenide thermoelectric systems [19]. These findings suggest that carefully designed multi-element lead chalcogenide alloys could potentially achieve ultra-low thermal conductivity while maintaining favorable electronic transport properties and addressing sustainability concerns [20,21].

This study examines the thermoelectric properties of (Pb_0.8_Ge_0.2_Te)_0.95-*x*_(PbSe)_0.05_(PbS)_*x*_ alloys. It has the purpose of optimizing composition to achieve ultra-low thermal conductivity while preserving electronic transport performance. Phonon-scattering strategies can suppress the lattice component of thermal conductivity; however, maintaining high electrical conductivity (*σ*) is equally crucial. Because the electrical output of a thermoelectric device scales with the power factor (*σS*^2^), any deterioration in σ increases Joule heating. It then ultimately lowers the dimensionless figure of merit (*ZT*) even when the Seebeck coefficient remains large. Effective materials design must therefore strike a balance—blocking heat-carrying phonons without unduly hindering charge-carrier mobility. By judiciously introducing selenium and sulfur to reduce tellurium content and by exploring compositions that minimize lead usage, this work addresses both resource scarcity and environmental impact. Through the precise control of phase segregation, we realize exceptionally low thermal conductivity values of 0.6–1.1 Wm^−1^K^−1^. The values elucidate the key structure–property relationships that govern performance in these multi-component systems, paving the way for next-generation sustainable thermoelectric materials.

## 2. Materials and Methods

### 2.1. Material Synthesis and Sample Preparation

The synthesis of (Pb_0.8_Ge_0.2_Te)_0.95-_*_x_*(PbSe)_0.05_(PbS)*_x_* alloys was carried out using a systematic three-step approach designed to minimize volatile element loss and ensure compositional homogeneity. This multi-stage process involves the initial preparation of binary chalcogenide precursors, followed by controlled melting with additional elements and final consolidation through hot-press sintering.

Step 1—Preparation of PbSe and PbS Precursors

High-purity Pb (99.999%), Se (99.999%), and S (99.99%) are weighed in an exact 1:1 stoichiometric ratio to form PbSe and PbS, respectively. The elemental mixtures are placed in quartz ampoules, evacuated to pressures under 10^−4^ torr, flame sealed, and melted at 1150 °C for 10 h. The high vacuum eliminates oxidation and suppresses Se and S volatilization, producing single-phase homogeneous PbSe and PbS ingots after slow cooling.

Step 2—Synthesis of Target Composition

The pre-synthesized PbSe and PbS are blended with elemental Pb, Ge, Te, and Na to synthesize polycrystalline samples with the actual composition of Na-doped (Pb_0.78_Na_0.02_Ge_0.2_Te)_0.95-_*_x_*(PbSe)_0.05_(PbS)*_x_*, which will be denoted hereinafter as (Pb_0.8_Ge_0.2_Te)_0.95-_*_x_*(PbSe)_0.05_(PbS)*_x_* for simplicity. Sodium substitutes at Pb sites, creating acceptor levels that raise hole concentration while preserving structural integrity. The powder mixture is sealed in high-vacuum ampoules under 10^−4^ torr and melted at 1150 °C for 10 h to ensure full homogenization. Immediately after melting, the ampoules are rapidly quenched in water, followed by annealing at 500 °C for 72 h [3,14].

Step 3—Powder Processing and Hot Press Consolidation

The quenched ingots are crushed to a powder, loaded into a 12.7 mm diameter graphite die, and hot-pressed at 500 °C under a uniaxial pressure of 40 MPa for 1 h in vacuum. Archimedes’ measurements show final bulk densities of 7.50–7.70 g/cm.

### 2.2. Structural and Transport Property Characterization

Crystallographic phase analysis and lattice parameter determination were conducted using powder X-ray diffraction (XRD) with a Cu Kα radiation source (D8 Advance, Bruker, Berlin, Germany). The diffraction patterns were collected over appropriate angular ranges to identify phase purity and determine structural parameters through Rietveld refinement analysis.

The Seebeck coefficient and electrical conductivity were simultaneously determined using four-probe measurement methodology employing a commercial thermoelectric characterization system (ZEM-3, ULVAC, Chigasaki, Japan). Temperature-dependent thermal conductivity was calculated using the thermal conductivity relationship [3,14]:

(2) $$\kappa_{T}=\rho \lambda C_{P}$$

where *ρ*_s_ represents sample density measured by the Archimedes principle, *λ* is thermal diffusivity obtained through laser flash analysis (LFA-447, NETZSCH, Selb, Germany), and *C_P_* is the specific heat capacity determined using the Dulong–Petit approximation. The dimensionless figure of merit *ZT* was subsequently calculated from the measured transport parameters using the standard relationship.

## 3. Results and Discussion

**Figure 1:**
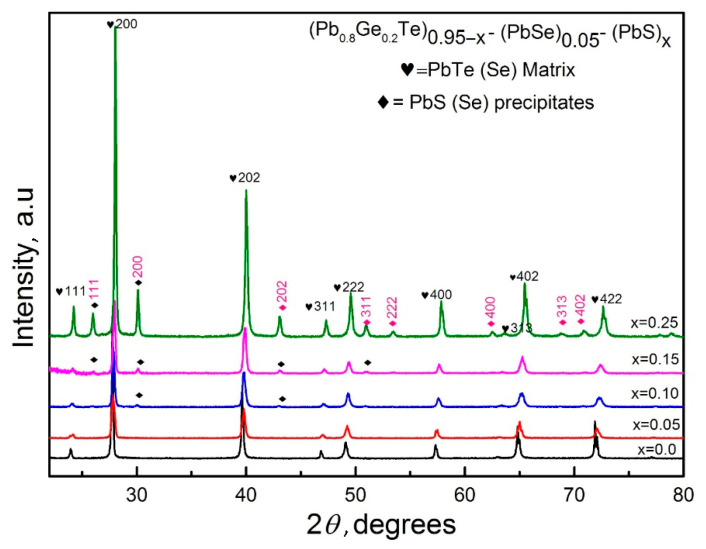
XRD patterns of (Pb_0.8_Ge_0.2_Te)_0.95-_*_x_*(PbSe)_0.05_(PbS)*_x_* alloys showing matrix phase (♥) and secondary precipitate (♦) evolution with PbS substitution.

Figure 1 presents the X-ray diffraction patterns of (Pb_0.8_Ge_0.2_Te)_0.95-_*_x_*(PbSe)_0.05_(PbS)*_x_* samples across varying PbS concentrations (*x* = 0.00 to 0.25), revealing significant structural transformations with compositional changes. The dominant peaks correspond to the cubic PbTe–Se matrix phase (marked with hearts), which is consistent with the rock salt structure. Additional peaks attributed to PbS–Se precipitates (marked with diamonds) emerge progressively with increasing PbS content.

The indexed reflections at (111), (200), (202), (311), (222), (400), (402), and (422) planes confirm the maintenance of the cubic crystal structure for the dominant matrix phase throughout the compositional range. However, the appearance and intensification of secondary phase peaks at higher *x* values indicate that the solubility limit of PbS in the host matrix has been exceeded, leading to phase segregation. This structural evolution suggests a transition from a single-phase solid solution at low PbS concentrations to a multiphase system at higher substitution levels, a phenomenon commonly observed in similar systems [22].

**Figure 2:**
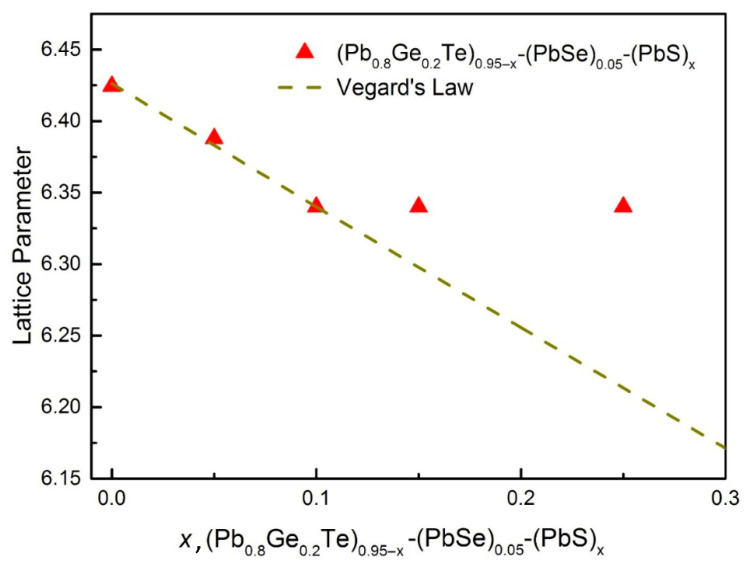
Compositional dependence of lattice parameter (Pb_0.8_Ge_0.2_Te)_0.95-*x*_(PbSe)_0.05_(PbS)*_x_* alloys compared with Vegard’s law prediction.

The lattice parameter analysis in Figure 2 provides quantitative confirmation of the structural changes observed in the XRD patterns, demonstrating a clear correlation between compositional variation and crystal lattice modifications. Initially, the lattice parameter decreases linearly with increasing PbS content, following Vegard’s law and reflecting the successful incorporation of smaller sulfur atoms into the crystal lattice. However, beyond *x* = 0.10, the experimental values deviate significantly from the theoretical prediction, plateauing at approximately 6.34 Å. This deviation coincides precisely with the emergence of secondary phases observed in Figure 1. It confirms that the solubility limit has been reached and excess PbS forms separate precipitate phases rather than being incorporated into the matrix lattice. The combined analysis reveals that while limited PbS substitution (*x* ≤ 0.10) results in lattice contraction and potential strain engineering, higher concentrations lead to hierarchical microstructures with coexisting phases that may be beneficial for thermoelectric applications through enhanced phonon scattering mechanisms [9].

**Figure 3:**
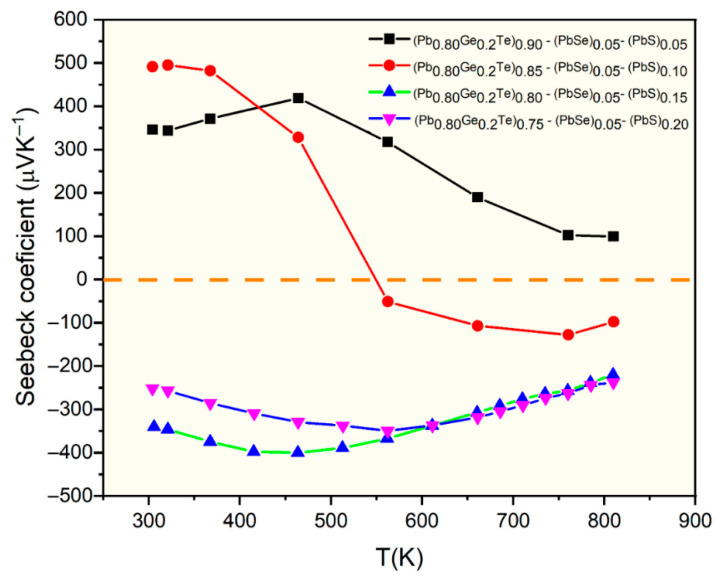
Thermoelectric power (Seebeck coefficient) as a function of temperature for (Pb_0.8_Ge_0.2_Te)_0.95-_*_x_*(PbSe)_0.05_(PbS)*_x_* alloys showing p-type to n-type transition with PbS content.

Figure 3 shows the temperature-dependent Seebeck coefficient of (Pb_0.8_Ge_0.2_Te)_0.95-*x*_(PbSe)_0.05_(PbS)*_x_* samples, offering critical insights into their carrier transport mechanisms and electronic band structure. The sign of the Seebeck coefficient directly indicates the dominant charge carrier type, with positive values for p-type (hole-dominated) and negative values for n-type (electron-dominated) conduction. A notable observation is the distinct carrier-type behavior across different PbS concentrations. For the sample with *x* = 0.05, the Seebeck coefficient remains consistently positive throughout the entire measured temperature range (300 K to 800 K), with values reaching approximately 500 μV/K at lower temperatures and decreasing to around 100 μV/K at higher temperatures. This unequivocally confirms that the *x* = 0.05 sample exhibits exclusive p-type behavior. It suggests that the pristine (Pb_0.8_Ge_0.2_Te)_0.95_(PbSe)_0.05_ matrix is inherently p-type, likely due to native acceptor defects such as Pb vacancies or slight stoichiometry deviations.

In stark contrast, samples with higher PbS content (*x* = 0.15 and *x* = 0.20) consistently display n-type behavior (negative Seebeck coefficient) across the entire measured temperature range, with absolute values ranging from 200 to 400 μV/K. This systematic transition from p-type (at *x* = 0.05) to n-type (at *x* = 0.15 and *x* = 0.20) with increasing PbS concentration is a strong indication that sulfur (S) acts as an electron donor in this PbTe(Se) system. The systematic transition from p- to n-type conduction with increasing S content is attributed to donor-like sulfur substituting for Te, which pushes the Fermi level closer to the conduction band minimum. While secondary PbS precipitates are present, our data does not imply that they directly control the Fermi-level position. However, they act mainly as additional phonon-scattering. This suggests that sulfur substitution and/or the presence of PbS phases either introduce additional electrons or suppress the native acceptor defects that otherwise cause p-type conduction [23].

Furthermore, the *x* = 0.10 sample exhibits particularly intriguing temperature-dependent behavior. At lower temperatures (up to approximately 500 K), this sample initially shows p-type characteristics with a positive Seebeck coefficient. However, as the temperature increases further (from about 500 K to 550 K), the Seebeck coefficient sharply drops, crosses the zero line, and becomes significantly negative at higher temperatures. This dramatic sign changes from positive (p-type) to negative (n-type) with increasing temperature, which is a hallmark of bipolar conduction. At elevated temperatures, the thermal generation of electron–hole pairs across the band gap becomes significant. While holes dominate at lower temperatures, the contribution of electrons becomes increasingly substantial (possibly due to a higher mobility or density of states). It eventually surpasses those holes, leading to a net negative Seebeck coefficient. This specific behavior for the *x* = 0.10 sample is precisely at the threshold of PbS solubility and precipitate formation. This suggests an optimal electronic environment where both carrier types contribute significantly to transport at elevated temperatures. The ability to control the carrier type by varying PbS content, coupled with the observation of bipolar conduction, provides crucial insights for engineering the electronic structure to optimize thermoelectric performance.

**Figure 4:**
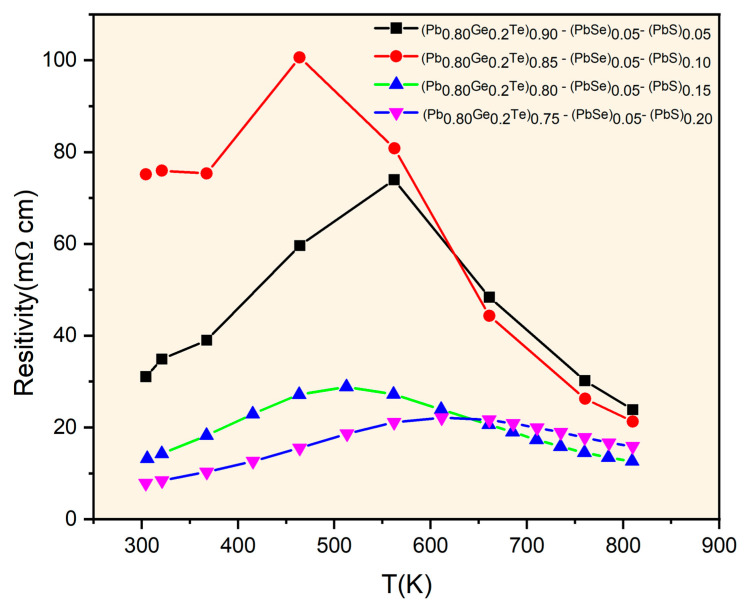
Resistivity as a function of temperature for (Pb_0.8_Ge_0.2_Te)_0.95-_*_x_*(PbSe)_0.05_(PbS)*_x_*.

Figure 4 illustrates the temperature-dependent electrical resistivity of the (Pb_0.8_Ge_0.2_Te)_0.95-*x*_(PbSe)_0.05_(PbS)*_x_* samples, revealing complex transport characteristics influenced significantly by both compositional changes and temperature. The resistivity values, ranging from approximately 10 to 100 mΩ∙cm, are notably higher than those typically observed in pristine PbTe-based materials [6]. It proposes enhanced carrier scattering due to the multi-element alloying and observed phase segregation. Samples with lower PbS content (*x* = 0.05 and *x* = 0.10) exhibit characteristic semiconducting behavior, where resistivity initially increases with temperature reaching peak values around 500–600 K (e.g., approximately 75 mΩ∙cm for *x* = 0.05 and 100 mΩ∙cm for *x* = 0.10). It is then followed by a distinct decrease at higher temperatures. This behavior, particularly the resistivity peak, aligns remarkably well with the onset of bipolar conduction, as indicated by the Seebeck coefficient analysis in Figure 3, where the *x* = 0.10 sample undergoes a transition from p-type to n-type at similar temperatures. The thermally activated generation of minority carriers (electrons in the *x* = 0.05 case or holes then electrons for *x* = 0.10) at elevated temperatures significantly increases the total carrier concentration, leading to the observed reduction in resistivity. In contrast, samples with higher PbS concentrations (*x* = 0.15 and *x* = 0.20) demonstrate considerably lower and more temperature-stable resistivity values, generally ranging from 10 to 25 mΩ·cm. This improved electrical stability and lower resistivity directly correlate with their consistently n-type character observed in Figure 3. This suggests that the increased PbS content, particularly when it leads to the formation of precipitates, optimizes electron transport by increasing carrier concentration and/or enhancing electron mobility relative to hole transport. The elevated overall resistivity across all samples is primarily attributed to multiple scattering mechanisms. It includes point defect scattering from compositional disorders, grain boundary scattering from phase segregation, and crucially, interface scattering between the matrix and the PbS precipitate phases identified in the structural analysis (Figure 1 and Figure 2). This intricate interplay between structural modifications, carrier-type control, and scattering mechanisms is fundamental to understanding and further optimizing the thermoelectric performance of these complex materials.

**Figure 5:**
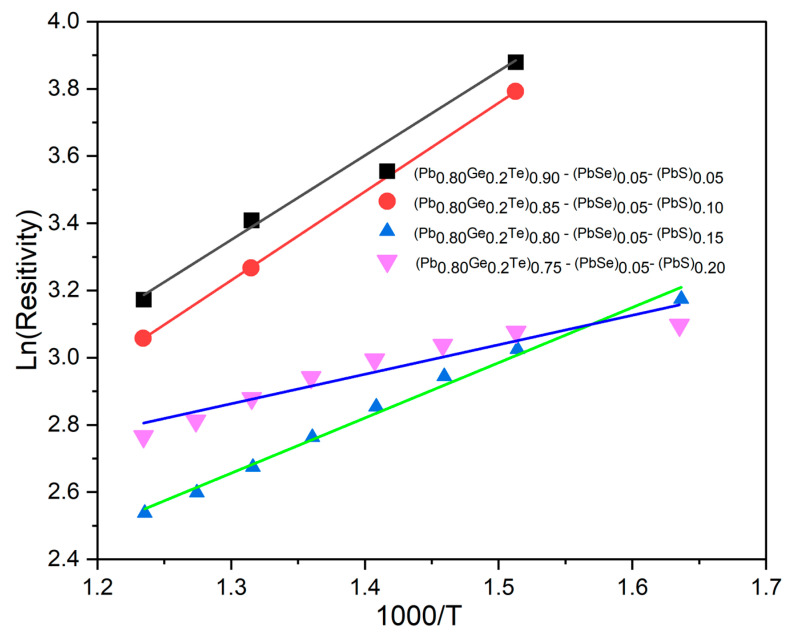
Arrhenius plots of electrical resistivity for Pb_0.8_Ge_0.2_Te)_0.95-_*_x_*(PbSe)_0.05_(PbS)*_x_*, the quaternary system in the high-temperature intrinsic regime (600–800 K).

To gain deeper insight into the charge transport mechanism, we analyzed the high-temperature (intrinsic) segment of the *ρ*(*T*) curves. Figure 5 displays Arrhenius plots of ln *ρ* versus 1000/*T* for all compositions. Above ≈ 600 K for each sample follows a straight line trend, confirming thermally activated conduction. The linear region was fitted with

(3) $$\ln\left(\rho \right)=\ln\left(\rho_{0}\right)+\frac{E_{g}}{2K_{B}}\;\frac{1000}{T}$$

where the slope (*E_g_*/2*k*) gives one-half of the optical band gap *E_g_*.

The extracted gaps decrease systematically from 0.50 eV (*x* = 0.05) to 0.15 eV (*x* = 0.20) (Table 1), reflecting band convergence and the donor-like nature of S and Te defects. This monotonic narrowing corroborates the p → n conduction switch observed in the Seebeck data and confirms that sulfur incorporation simultaneously tailors both carrier concentration and electronic structure.

**Figure 6:**
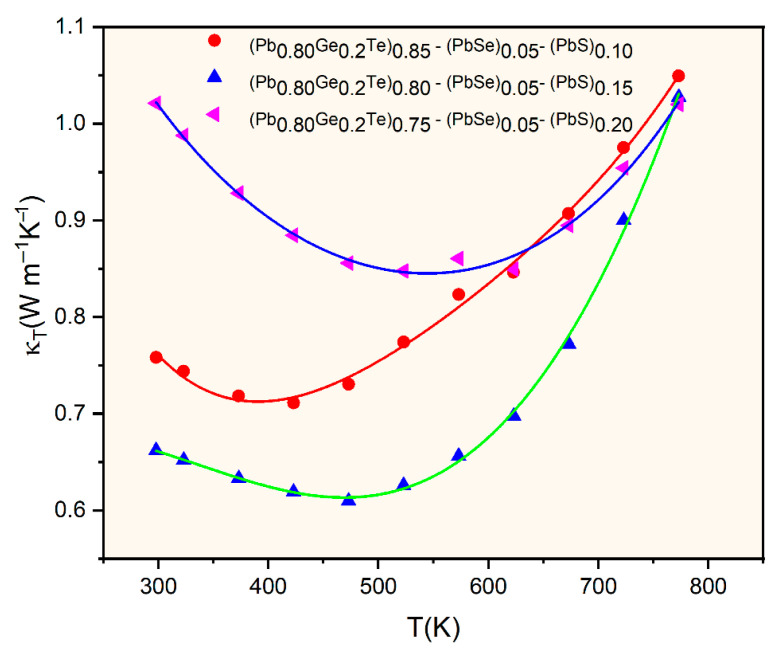
Temperature-dependent thermal conductivity of alloys with varying PbS contents (*x* = 0.10, 0.15, and 0.20). The solid lines represent fitted curves connecting experimental data points to guide the eye and illustrate temperature-dependent trends for each composition, with *x* = 0.10 (red), *x* = 0.15 (blue), and *x* = 0.20 (green).

Figure 6 presents the temperature-dependent total thermal conductivity (*κ_T_*) for the selected (Pb_0.8_Ge_0.2_Te)_0.95-*x*_(PbSe)_0.05_(PbS)*_x_* samples. It reveals remarkably low values ranging from approximately 0.6 to 1.1 W⋅m^−1^K^−1^ across the entire measured temperature range. These exceptionally low values represent a significant reduction compared to pristine PbTe (typically 2–3 Wm^−1^K^−1^). This indicates highly effective phonon scattering mechanisms within the engineered microstructure [3,14]. *κ_T_* initially decreases, reaching minimum values around 450–550 K, and subsequently increases at higher temperatures. The initial decrease is primarily attributed to enhanced Umklapp scattering (phonon–phonon scattering) with rising temperature, leading to a reduction in the phonon mean free path. The subsequent increase in *κ_T_* at higher temperatures is predominantly due to the onset of bipolar thermal conduction (*κ*_bipolar_). Thermally generated electron–hole pairs diffuse and contribute to heat transport, a phenomenon consistent with the Seebeck coefficient behavior observed in Figure 3. The compositional dependence of thermal conductivity is evident, with *κ_T_* generally decreasing as the PbS content (*x*) increases from 0.10 to 0.20. Notably, the *x* = 0.15 sample exhibits the lowest overall thermal conductivity across most of the temperature range, reaching approximately 0.6 Wm^−1^K^−1^ at lower temperatures. This ultra-low thermal conductivity primarily results from comprehensive phonon scattering mechanisms operating across multiple length scales. It is the hallmark of the hierarchical microstructure engineered through compositional design. At the atomic scale, the significant mass contrast between Pb, Ge, Te, Se, and S atoms creates potent point defect scattering.

**Figure 7:**
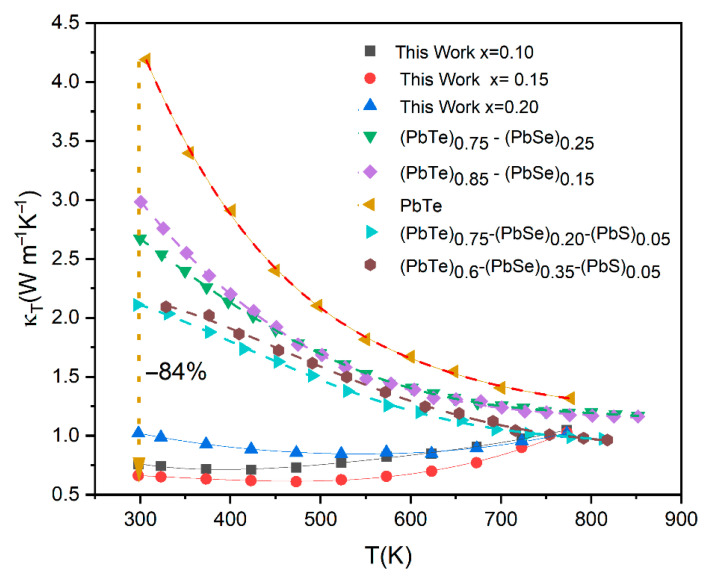
Comparing total thermal conductivity of (Pb_0.8_Ge_0.2_Te)_0.95-*x*_(PbSe)_0.05_(PbS)*_x_* with PbTe, (PbTe)_0.85_-(PbSe)_0.15_ [7], (PbTe)_0.75_-(PbSe)_0.25_ [7], (PbTe)_0.75_-(PbSe)_0.20_-(PbS)_0.05_ [3,14], and (PbTe)_0.6_-(PbSe)_0.35_-(PbS)_0.05_ [3,14].

Figure 7 illustrates a comprehensive comparison of the temperature-dependent total thermal conductivity (*κ_T_*) of the current work’s (Pb_0.8_Ge_0.2_Te)_0.95-*x*_(PbSe)_0.05_(PbS)*_x_* samples (*x* = 0.10, 0.15, 0.20) against various PbTe-based materials reported in the literature, including pristine PbTe, (PbTe)_0.75_-(PbSe)_0.25_ [7], (PbTe)_0.85_-(PbSe)_0.15_ [7], (PbTe)_0.75_-(PbSe)_0.20_-(PbS)_0.05_ [3,14], and (PbTe)_0.6_-(PbSe)_0.35_-(PbS)_0.05_ [3,14].

This comparison rigorously validates the effectiveness of the current compositional engineering strategy in achieving ultra-low thermal conductivities. The most striking observation from Figure 6 is the remarkably lower *κ_T_* values achieved in the current work compared to all other reference materials across the entire measured temperature range. Pristine PbTe (yellow inverted triangles) exhibits significantly high thermal conductivity, starting above 4.0 Wm^−1^K^−1^ at room temperature and gradually decreasing. Compared to this, the samples from the present study demonstrate a dramatic reduction, with the lowest value for our *x* = 0.15 sample (red circles) being around 0.6 Wm^−1^K^−1^ at its minimum, representing an exceptional reduction of over 8% (specifically, approximately 84% relative to pristine PbTe at 300 K). This demonstrates the specific combination of Ge, Se, and particularly the controlled introduction of PbS leading to phase segregation in the (Pb_0.8_Ge_0.2_Te)_0.95-*x*_(PbSe)_0.05_(PbS)*_x_* system. It is highly effective in suppressing phonon transport and further augmented by lattice strain induced by size mismatch, which collectively disrupts phonon propagation. Moreover, the formation of secondary phases and precipitates, specifically PbS(Se) (as unambiguously confirmed by the XRD analysis in Figure 1 and the deviation from Vegard’s law in Figure 2), introduces abundant nanoscale interfaces that effectively scatter mid-frequency phonons. Simultaneously, grain boundaries provide additional scattering centers for longer-wavelength phonons [8]. The achievement of such extremely low thermal conductivity, approaching the amorphous limit (often termed “glass-like” thermal conductivity) while largely preserving the crystalline electronic structure, represents an ideal scenario for thermoelectric materials, as it enables the decoupling of thermal and electrical transport [13].

**Figure 8:**
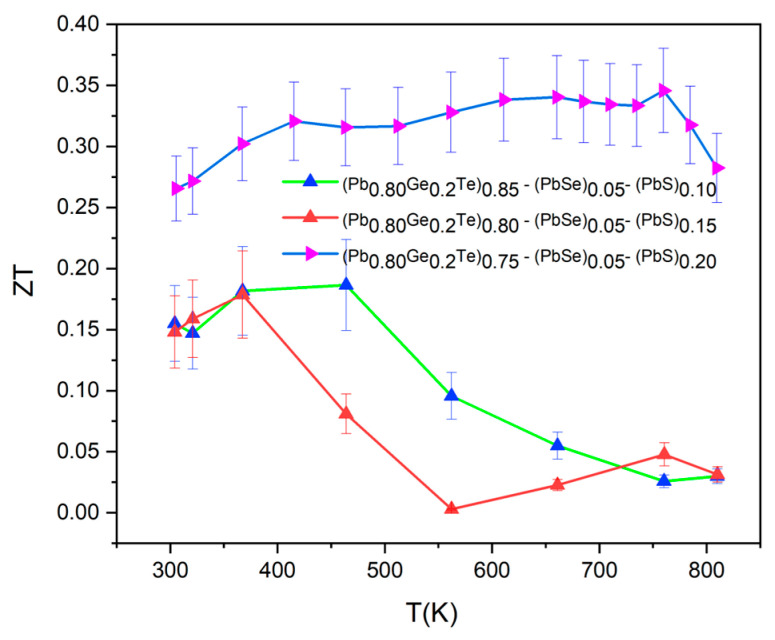
Compositional optimization of thermoelectric performance in (Pb_0.8_Ge_0.2_Te)_0.85-_*_x_*(PbSe)_0.05_(PbS)*_x_* system.

Figure 8 presents the temperature-dependent thermoelectric figure of merit (*ZT*) for (Pb_0.8_Ge_0.2_Te)_0.95-*x*_(PbSe)_0.05_(PbS)*_x_* samples, which encapsulates the synergistic effects of the individual transport properties (*S*, *σ*, and *κ_T_*). The *ZT* values were calculated using the relationship *ZT* = *S*^2^*σT*/*κ_T_*, where *S* is the Seebeck coefficient, *σ* is the electrical conductivity (inverse of resistivity), and *κ_T_* is the total thermal conductivity [24].

Despite the successful reduction in thermal conductivity as shown in Figure 6, the overall *ZT* values remain relatively low, with a maximum value of approximately 0.34 achieved for the *x* = 0.20 sample at around 650 K. This peak *ZT* value is significantly lower than that typically reported for optimized PbTe-based materials, which can often reach *ZT* values of ~1.0–2.0 or even higher. The reasons for this comparatively lower *ZT* in the current system are multifaceted and can be understood by critically examining the interplay of the individual transport parameters. While the thermal conductivity has been effectively suppressed, the electrical power factor (*S*^2^*σ*) appears to be the primary limiting factor for achieving higher *ZT* values, at *x* = 0.10 and *x* = 0.15 samples. Their *ZT* values are moderate, with peaks around 0.15–0.18 at 350–400 K, but their performance deteriorates significantly at higher temperatures. This sharp decline in *ZT* at elevated temperatures for these compositions is primarily attributed to the prominent bipolar conduction. The corresponding increase in both electrical resistivity (as carrier type shifts or becomes mixed) and total thermal conductivity (*κ_T_*) due to bipolar contribution can be observed in Figure 3, Figure 4 and Figure 5. Although the *x* = 0.20 sample shows the highest *ZT* of 0.34 at 650 K and maintains values above 0.30 from 500 to 700 K, suggesting an optimal balance of properties at this composition [7,25], its absolute value is still limited by the overall relatively low power factor. While the structural analysis confirmed the successful engineering of a hierarchical microstructure and enhancing phonon scattering, further optimization is needed to reduce carrier scattering. It is also expected to improve electrical conductivity without negatively impacting the Seebeck coefficient or increasing thermal conductivity. Achieving higher *ZT* values in these complex multi-element systems necessitates a delicate balance and fine-tuning of carrier concentration and mobility, which remains a key challenge for future research.

## 4. Conclusions

This systematic investigation demonstrates successful engineering of ultra-low thermal conductivity (0.6–1.1 Wm^−1^K^−1^) in (Pb_0.8_Ge_0.2_Te)_0.95-_*_x_*(PbSe)_0.05_(PbS)_x_ alloys through controlled multi-element alloying and hierarchical microstructure development. The optimal *x* = 0.20 composition, corresponding to (Pb_0.8_Ge_0.2_Te)_0.75_(PbSe)_0.05_(PbS)_0.20_, achieved a peak *ZT* value of 0.34 at 650 K while reducing tellurium content by 25%. Remarkably, at *x* = 0.15 corresponding to (Pb_0.8_Ge_0.2_Te)_0.80_(PbSe)_0.05_(PbS)_0.15_, thermal conductivity reached 0.6 Wm^−1^K^−1^, approaching glass-like thermal conductivity values. Structural analysis revealed critical solubility thresholds where PbS substitution transitions from a solid solution to controlled phase segregation at *x* > 0.10. It creates multi-scale phonon scattering that suppresses thermal conductivity by 84% compared to pristine PbTe. The primary novelty lies in demonstrating controlled phase segregation for ultra-low thermal conductivity while addressing tellurium scarcity through strategic sulfur incorporation. The successful decoupling of thermal and electrical transport validates the “phonon-glass electron-crystal” concept, establishing fundamental principles for next-generation thermoelectric materials. These findings provide a promising pathway for scalable cost-effective energy conversion technologies with significant potential for waste heat recovery applications in automotive, industrial, and power generation sectors.

## Figures and Tables

**Table 1:** Intrinsic band-gap energies E_g_ of (Pb_0.80_Ge_0.2_Te)_0.95-_*_x_*(PbSe)_0.05_(PbS)*_x_* alloys, obtained from Arrhenius fits of ln *ρ* versus 1000/*T* in the 600–800 K.

*x* in (Pb_0.80_Ge_0.2_Te)_0.95-_*_x_*(PbSe)_0.05_(PbS)*_x_*	Slope (ln *ρ* vs. 1000/*T*)	*E_g_*
0.05	2.510	0.433
0.10	2.640	0.455
0.15	1.64	0.283
0.20	0.877	0.151

## Data Availability

The original contributions presented in the study are included in the article; further inquiries can be directed to the corresponding authors.
